# Removal of Chromium from a Contaminated Soil Using Oxalic Acid, Citric Acid, and Hydrochloric Acid: Dynamics, Mechanisms, and Concomitant Removal of Non-Targeted Metals

**DOI:** 10.3390/ijerph16152771

**Published:** 2019-08-02

**Authors:** Yuhuan Sun, Feng Guan, Weiwei Yang, Fayuan Wang

**Affiliations:** 1 College of Environment and Safety Engineering, Qingdao University of Science and Technology, Qingdao 266042, China; 2 Yantai Institute of Coastal Zone Research, Chinese Academy of Sciences, Yantai 264003, China; 3 Key Laboratory of Soil Resources and Environment in Qianbei of Guizhou Province, Zunyi Normal University, Zunyi 563002, China

**Keywords:** chemical leaching, soil washing, column experiment, chromium, soil remediation

## Abstract

Soil leaching is an effective remediation technique using agents to leach the target pollutants from the soil. However, the dynamics and mechanisms for leaching of Cr and other non-pollutant metals from Cr-contaminated soils are not yet well understood. Here, column leaching experiments were conducted to determine the effect of hydrochloric acid (HCl), citric acid (CA), and oxalic acid (OX) on the leaching of Cr, as well as of Ca, Mg, Fe, and Mn, from a soil contaminated by a Cr slag heap. Acid leaching decreased soil pH and enhanced the mobility of all the surveyed metals. Leaching dynamics varied with both metals and acids. OX had the highest removal rates for Cr, Fe, Mn, and Mg, but had the poorest ability to leach Ca. HCl leached the largest amount of Ca, while CA leached similar amounts of Mg and Mn to OX, and similar amounts of Fe and Cr to HCl. Cr in the leachates was correlated with Ca, Mg, Fe, and Mn. Cr mainly interacted with soil mineral components and showed a punctate distribution in soil particles. The X-ray diffraction (XRD), scanning electron microscopy with energy-dispersive X-ray spectroscopy (SEM-EDS), and X-ray photoelectron spectroscopy (XPS) analyses showed soil mineralogical and morphological properties were differently altered after leaching by different acids. Complexation of Cr(III), competitive desorption, and reduction of Cr(VI) make significant contribution to Cr leaching by organic acids. In conclusion, OX can be applied in leaching remediation of Cr-contaminated soil, but the concomitant removal of other non-targeted metals should be taken into account because of the loss of soil minerals and fertility.

## 1. Introduction

Chromium (Cr) and its compounds have various applications in industrial processes, such as alloy production, electroplating, wood preservation, leather processing, development of pigments, printing and dyeing, and catalysis [1]. Like many other transition metal elements, Cr is essential to humans at low concentrations but toxic at higher concentrations, and thus considered a potential contaminant in soil and groundwater [2]. Cr can enter the environment by human activities (such as mining and plating) and several natural processes (such as weathering and biochemical reactions), and can subsequently cause environmental pollution [1,3,4], posing potential environmental risks. Thus, the efforts should be undertaken on effective remediation for Cr-contaminated sites.

Soil leaching (or washing) is a widely used technique for soil remediation, which uses agents, such as inorganic eluent, chelation agents, and surfactant, to leach the target pollutants from the soil [5]. Acids are commonly used as eluents, because they can dissolve insoluble compounds such as carbonates and oxides, and disassociate metals from soil surfaces, thus enhancing their extractability and removal from soil [6,7]. Different acids have been applied in soil leaching, including inorganic acids such as hydrochloric acid (HCl), H_2_SO_4_, HNO_3_, H_3_PO_4_, and H_2_SiF_6_, and organic acids such as formic acid, acetic acid, oxalic acid (OX), citric acid (CA), tartaric acid, and polyglutamic acid [8,9]. The metal removal efficiency not only depends on acids and metals contamination characteristics, but also varies with soil physicochemical properties such as soil pH, texture, cation exchange capacity (CEC), and organic matter (OM) characteristics [8]. Therefore, more case studies are needed to enlarge the applicability of acid leaching.

Since acids are generally non-selective, they can leach out not only the pollutants, but also other elements including essential nutrients for organisms, inevitably causing soil matrix dissolution and poor soil structure, and the decrease in soil pH and fertility. Evidently, an ideal extractant should be environment-friendly, and can remove pollutants as much as possible, but remove non-pollutant elements as little as possible. During the leaching process of Cr and Ni using ethylenediaminetetraacetic acid (EDTA) and CA, other elements such as Ca, Fe, Mg, Al, Mn, and Zn were also dissolved [10]. In another study, CA and tartarate (TA) were found as effective as EDTA and diethylenetriaminepentaacetic acid (DTPA) to extract pollutants (Cd, Cu, Pb, and Zn) from two contaminated soils, but they removed 80% less soil macronutrients (Fe, Ca, and Mg) and improved soil structure [11]. Thus, different agents may have various impacts on soil characteristics. However, the remediation mechanisms of target pollutant and concomitant removal of non-targeted metals during the remediation process are not yet well recognized.

Among the commonly used leaching agents, HCl could effectively dissolve minerals and transport metals and complexes in soil [6]. Organic acids such as CA and OX are considered biodegradable and environment-friendly low molecular weight organic acids [10,12,13], which may exert less adverse influences on soil quality. Differently to HCl, organic acids CA and OX can act as reducers and chelates of toxic metals, such as Cr [14]. Inorganic and organic acids may have different removal mechanisms during Cr leaching, and concomitant effects on leaching of non-targeted elements. Here, for the first time, the remediation effects and mechanisms of HCl, CA, and OX on a Cr-polluted soil were compared using column leaching experiment. Our aims are (1) to compare the dynamics of Cr concentrations in the soil leachates with different acids; (2) to test the removal of non-targeted metals by the acids; and (3) to elucidate the mechanisms of Cr leaching via investigating the mineralogical and morphological properties of the soils before and after the leaching. The results will help to further recognize the leaching remediation mechanisms of Cr-polluted soil.

## 2. Materials and Methods

### 2.1. Soil

The contaminated soil used for experiments was collected from the upper 20 cm surface layer at a Cr-containing slag heap site of Qingdao Hongxing Chemical Plant, China (longitude 120°23′18″ E, latitude 36°12′21″ N). Five samples (2 kg per sample) were randomly collected from different locations, and then mixed thoroughly after returning to the laboratory. For soil analyses and column leaching experiment, the mixed samples were air-dried and sieved by a 2 mm sieve. The physical and chemical properties of the soil are shown in Table 1.

### 2.2. Column Leaching Experiment

**Figure 1:**
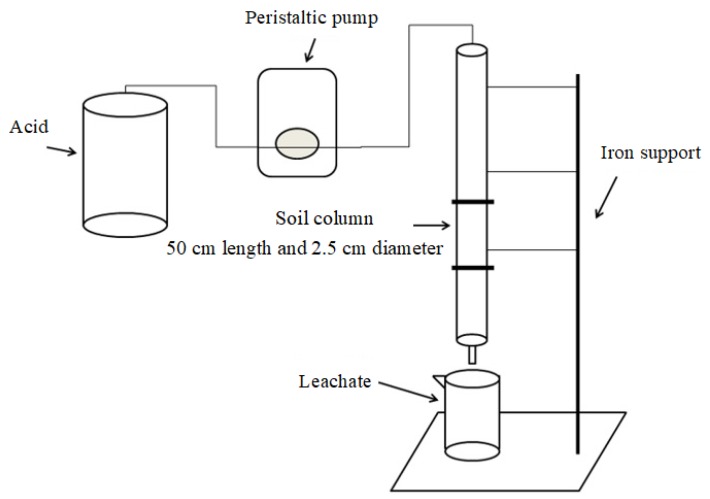
Diagram of the leaching device.

The diagram of the leaching device is shown in Figure 1. The tube body of the soil column was made by plexiglass column with 50 cm length and an inner diameter of 2.5 cm. Each column was filled with 200 g Cr-contaminated soil, with a depth of about 30 cm. The bottom of the soil column was immersed in distilled water until the saturation equilibrium, based on the pre-determined saturated moisture content (40% wt. percentage). A 100 μm-aperture sand core was placed at the bottom of the column, and a 1–2 cm thick quartz layer was placed above the sand core and then loaded the soil into the column. Columns were leached continuously with 800 mL HCl, CA, or OX, respectively. Based on our pre-experiments, the concentrations of the acids were set as 0.5 mol/L. The eluents depend on the pressure of the peristaltic pump to enter the soil column uniformly, and the flow rate was 0.75–1.00 mL/min, which was based on our pre-experiments. A volume of 40 mL acid was added at each time. The next 40 mL acid was added after 40 mL leachate of the former 40 mL acid was collected (about 12 h) using a 50 mL beaker. The leachate was filtered into a 50 mL PE plastic bottle, and then pH value was determined immediately. The concentrations of Ca, Mg, Fe, Mn, and Cr in the leachate were determined using flame atomic absorption spectrometry (FAAS) (AA-7000, Shimadzu, Tokyo, Japan). After the leaching procedure, the soil in the column was air-dried and mixed thoroughly, and then sampled for further analysis.

### 2.3. Mineralogical and Morphological Analysis

Mineralogical characterization of soil samples was performed using an X-ray diffractometer (XRD) (Rigaku D/max-2500 PC, Rigaku Industrial Corp., Tokyo, Japan), operating with Cu Kα radiation at 40 kV and 150 mA, scanning over the range 10–90° in 2θ, step size 0.02°. To analyze the overall size distribution and morphology of soil particles, and elemental composition, a scanning electron microscope (SEM) (S-4800, Hitachi, Tokyo, Japan) equipped with X-ray energy dispersive spectrometer (EDS) was used. The elemental analysis by EDS was performed in “point mode” in which the beam was positioned on a manually selected single area on SEM image [15,16]. Chemical states of Cr on the surface of soil samples before and after leaching were determined using an ESCALAB 250Xi X-ray photoelectron spectroscopy (XPS) (Thermo Fisher Scientific, Waltham, USA) with an Al Kα X-ray source at a base pressure of 10^−9^  mbar. To compensate for surface charge effects, binding energies were calibrated using the C 1s hydrocarbon peak at 284.8 eV. A narrow scanned spectra in the range of 567–596 eV was used to obtain the redox state information for the Cr. Peak fitting procedure and quantitative calculations of the surface atomic concentration of Cr were performed using XPS Peak software (version 4.1).

### 2.4. Data Analysis

The XRD data were analyzed using Jade 7.1.2 software (Jade Software Corp., Christchurch, New Zealand). The data were analyzed using SPSS 21.0 statistical software and Microsoft Office Excel 2010. One-way analysis of variance (*p* < 0.05), followed by the Tukey test, was performed to determine statistical significance between the different treatments. The Pearson correlation test was performed to determine the correlation between Cr concentrations and other metal concentrations in leachates.

## 3. Result and Discussion

### 3.1. Leachate pH and Soil pH

**Figure 2:**
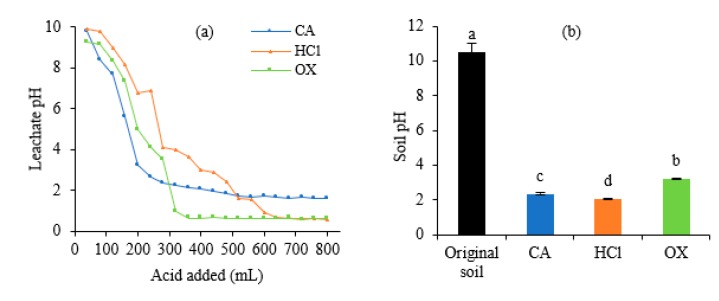
The pH values in the leachates and soils after leaching by different acids. (**a**) Leachates, (**b**) Soils. In Figure 2b, different letters on the bars indicate significant differences among the means in different treatments using a one-way analysis of variance followed by the Tukey test (*p* < 0.05).

The dynamic changes of the leachate pH were shown in Figure 2a. At the beginning of the leaching (120 mL), leachate was alkaline (>7.7). With the increase of the eluents volume, pH value showed a rapid decline. When the soil received 240 mL CA, 320 mL OX, and 640 mL HCl, leachate pH decreased to 2.64, 0.94, and 0.68, respectively, and then decreased slowly and stabilized at 1.6, 0.6, and 0.5 thereafter. After the leaching procedure, pH values of soil treated with HCl, CA, and OX decreased significantly (*p* < 0.05) to 2.07, 2.35, and 3.20, respectively (Figure 2b).

Soil pH influences chemical processes and chemical reactions of metals in soil, such as oxidation, reduction, precipitation, adsorption, and coordination reaction [17]. It was found that there are two main modes on the leaching rates of metals being enhanced by acids from soils. The first is that acids would produce positively charged hydrogen atoms, inducing a multi-stage proton by H^+^ or the replacement reaction of acid with metals [18]. The second is the dissolution reaction with the mineral crystal lattice of the soil by the H^+^ absorbed to the surface of minerals [6,19]. The present results show that acid leaching could decrease soil pH, which possibly increases the mobility of metals and then accelerates their leaching in the soil [20,21]. Among the three acids, OX caused the least soil acidification, which is of significance for re-use of the leached soil.

### 3.2. Leaching Dynamics

**Figure 3:**
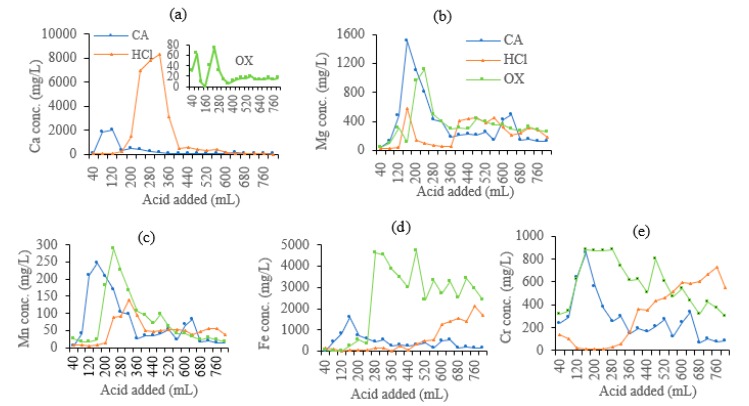
Dynamic concentrations of metals in leachates of the soils leached by different acids. (**a**) Ca, (**b**) Mg, (**c**) Mn, (**d**) Fe, (**e**) Cr.

**Figure 4:**
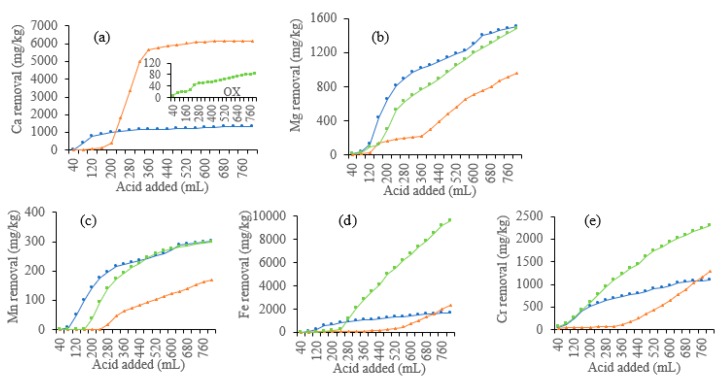
Cumulative removal of metals from the soils leached by different acids. (**a**) Ca, (**b**) Mg, (**c**) Mn, (**d**) Fe, (**e**) Cr.

As shown in Figure 3a, the leaching loss of Ca mainly occurred in the first half stage of the leaching. At the end of the leaching, the cumulative removal of Ca by HCl, CA, and OX was 6176, 1347, and 86 mg Ca/kg soil, respectively (Figure 4a). HCl leached much more Ca than CA and OX, while OX showed the least leaching effect. For CA, the peak value of leaching Ca was significantly earlier than the other two acids, indicating that Ca leaching by CA reaches the equilibrium state much sooner than leaching by the other two acids.

The dynamic leaching process of Mg and Mn was similar (Figure 3b,c). The leaching loss of Mg and Mn also occurred mainly in the first half leaching stage and decreased after reaching a summit value. The cumulative leaching amount of Mg and Mn gradually increased. CA and OX had similar cumulative leaching amounts of Mg (1512 mg/kg for CA, and 1483 mg/kg for OX) and Mn (301 mg/kg for CA, and 300 mg/kg for OX), which were much higher than the amounts leached by HCl (957 mg/kg Mg and 171 mg/kg Mn) (Figure 4b,c). As organic acids, OX and CA showed similar effects on the leaching loss of Mg and Mn, with a difference in the peak value of CA which appeared earlier than that of OX.

The dynamic leaching of Fe and Cr was shown in Figure 3d,e. OX had the most cumulative leaching amounts of Fe (9648 mg/kg) and Cr (2304 mg/kg) (Figure 4d,e). Cr concentration first peaked at 160 mL leachate, with 838 mg/L, while Fe concentration reached the first peak at 400 mL leachate, with 3448 mg/L. Then, Cr and Fe reached the second peak concentrations at 480 mL leachate, with 801 mg/L and 4697 mg/L, respectively. Fe and Cr dynamic concentrations reached peak values fastest in CA leachates, but CA showed the least cumulative leaching amounts (1667 mg/kg Fe and 1102 mg/kg Cr, respectively). Fe concentration in the leachate reached a peak and then decreased gradually. HCl also showed a poor ability to leach Fe (2345 mg/kg) and Cr (1282 mg/kg), with most of Fe and Cr being leached during the middle and late stages.

Acids are generally nonspecific agents which can leach diverse elements from soil. Soil washing with acids can lead to loss of the major component elements such as Cu, Zn, Al, Ca, Mg, Fe, and Mn, and alter or even destroy soil structures [10,22,23]. CA would lead to leaching of major elements such as Ca, Mg, Fe, Al, Mn and Zn, in addition to pollutants Cr and Ni [10,24]. Our present results confirm that HCl, OX, and CA showed different capacities to leach Cr out of the soil, as well as essential nutrients for plants, Ca, Mg, Fe, and Mn, indicating the dissolution of soil mineral nutrients by both inorganic and organic acids. Acids that can effectively leach toxic metals but not nutrients should be selected for their potential applications in soil washing.

Our results show that acids vary greatly in their ability to leach different metals, with different dynamics and cumulative amounts of different metals. From the point of view of the total metals leached, OX generally had the most remarkable ability to leach Cr, Fe, Mg, and Mn, but the poorest ability to leach Ca. OX can effectively leach Cr via forming water-soluble chromium oxalate [25]. Reductive and ligand-promoted dissolution mechanisms may explain the effective extraction of Cr, Fe, and Mn by OX [26], but calcium oxalate precipitation formed between OX and Ca may allow the retention of Ca in soil. HCl leached the largest amount of Ca. Among the acids used in the present study, HCl has the strongest acidity, and can dissolve crystal lattices of the minerals in soils, and thus calcium carbonate would be dissolved to soluble Ca^2+^ by HCl [6]. Overall, CA leached similar amounts of Mg and Mn to OX, and similar amounts of Fe and Cr to HCl.

However, the metal concentrations in the leachates depend on not only acids but also metal species. Generally, Fe, Ca, and Cr are easier to be leached than Mg and Mn (Figure 4). This is in accordance with the previous findings of Jean-Soro et al. [10]. The leaching of Mn may be explained by the less content (546 mg/kg) in soil, while such explanations cannot explain the leaching of Mg, which is about 7209 mg/kg in soil, much higher than that of Cr. There must be other mechanisms deserving further investigation.

As for the leaching dynamics, the concentrations of Ca, Mg, Mn, Fe, and Cr (except for the HCl leaching of Fe and Cr) in the leachates generally increased to a peak, and then decreased slowly in the first 400 mL leachates, which had no significant correlation with leachate pH. There are two or more peaks during the leaching of Mg and Mn, with the larger peak in the first half stage and the smaller peak in the second half stage. However, in the process of Fe and Cr leaching by HCl, the concentrations of these two metals in the leachate varied irregularly all the time and even higher in the final stage, indicating a significant lag effect.

Metals in soil are generally bound to different soil fractions with different bonding forces, which may determine their extractability. For example, some metal ions that associate with functional groups of soil organic matters via complexation reaction are difficult to be desorbed, while those electrostatically adsorbed on the charge of soil colloids are much easier to be desorbed [17,27,28]. Coordinate–covalent bonding mainly occurs in inner-sphere metal complexes, whereas electrostatic bonding dominates outer-sphere complexes, which determines that inner-sphere complexes are generally more stable than outer-sphere complexes [29]. Understandably, the rapidly desorbed ions will have a fast increase in concentration in leachates, while those specifically adsorbed metals are generally desorbed slowly, resulting in an increase in concentration followed by a slow release in the initial leaching process [17]. These findings can also explain our present results that the metals have different leaching dynamics.

### 3.3. Correlation Analysis

As shown in Figure S1 (see Supplementary file for additional information), in most cases, linear relationships occurred between Cr and other metals. Cr always correlated positively with Mg and correlated positively with Fe in CA and HCl leachates, and Mn in CA and OX leachates. It has been shown that Cr and Mn had a highly positive correlation, as iron–manganese-rich oxides on the surface of mafic minerals coexist with Cr [30]. Our earlier study also found that the proportion of oxidized Cr (Fe-Mn-bound forms) in the soil was higher than other speciation. After leaching, the proportion of the oxidized Cr decreased significantly (unpublished data).

In addition, Cr correlated positively with Ca in CA leachates, but negatively with Ca in HCl leachates, and not significantly with Ca in OX leachates. Cr and Ca in minerals had different chemical behaviors when different acids were used in leaching. Ca forms stable complexes with oxalate and citrate [31,32], but dissolves readily in strong acid such as HCl, which may partly explain the contradictory correlations between Cr and Ca in different leachates.

### 3.4. XRD Analysis

**Figure 5:**
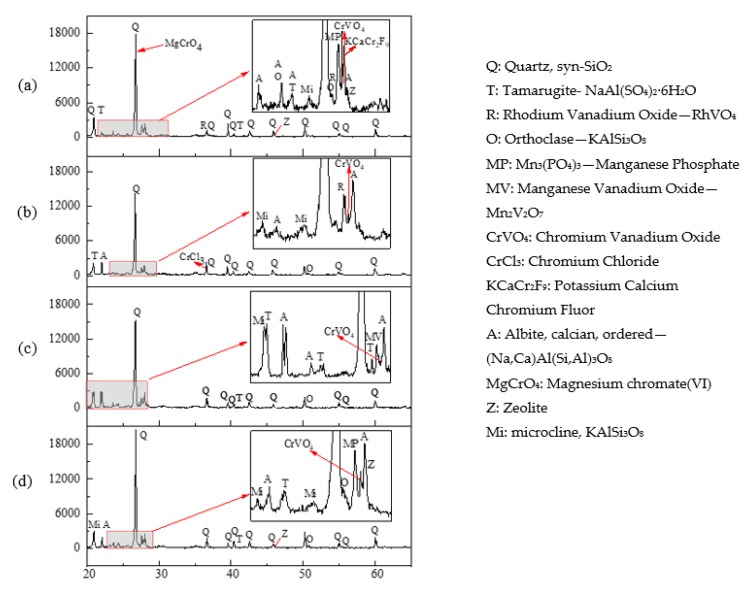
X-ray diffraction patterns of the soils before and after leaching by different acids. (**a**) Original soil, (**b**) soil leached by HCl, (**c**) soil leached by CA, (**d**) soil leached by OX.

The mineralogical components of the soils before and after leaching were analyzed by XRD (Figure 5). The original soil was characterized by the main mineral quartz, as well as other minor minerals, such as orthoclase, tamarugite, albite, microcline, zeolite and other silicate minerals (Figure 4a). Previous results have found several soil crystalline phases, particularly calcite, vermiculite and illite, can rapidly and fully dissolve under low soil pH conditions [33,34]. In our present study, on one hand, some diffraction peaks disappeared after leaching, suggesting the removal of some phases by the acids. On the other hand, some new crystalline phases generated as a result of the dissolution of acids, which was indicated by the appearance of the XRD peaks, especially albite and silicate-based minerals. Acid dissolution and metal ions complexing reactions of the organic acids may account for stimulating soil metal release and generation of new mineral (chromium chloride). Noteworthily, after leaching, the diffraction peaks of zeolite were observed only in OX-leached soil, suggesting this mineral was difficult to be decomposed by OX, and this can also explain the poor removal for Ca by OX.

XRD technique in combination with other synchrotron X-ray microanalytical analysis was used to confirm the geochemical forms (chromite and zincochromite) of Cr in a soil from an industrial polluted site [35]. In soils derived from fluvioglacial sands, Cr was predominantly in the fraction bound with iron and manganese oxides [36]. In the present study, XRD analysis showed that, the crystalline phases of the Cr-contained minerals detected in the original soil included magnesium chromate (VI) (MgCrO_4_), potassium calcium chromium (III) fluor (KCaCr_2_F_9_), and chromium (III) vanadium oxide (CrVO_4_), exhibiting strong peaks between 26–28°. Compared with the original soil, the peaks of MgCrO_4_ and KCaCr_2_F_9_ disappeared in all the leached soils. This is due to the facts that MgCrO_4_ and KCaCr_2_F_9_ are more easily decomposed and dissolved by acids. However, the insoluble CrVO_4_ was detected in the original and leached soils. In addition, in the soil after HCl leaching, chromium chloride (CrCl_3_) was also detected, which probably originated from the combination of residual Cl^−^ and Cr(III). No other Cr-minerals were observed in the XRD results.

### 3.5. SEM-EDS Analysis

**Figure 6:**
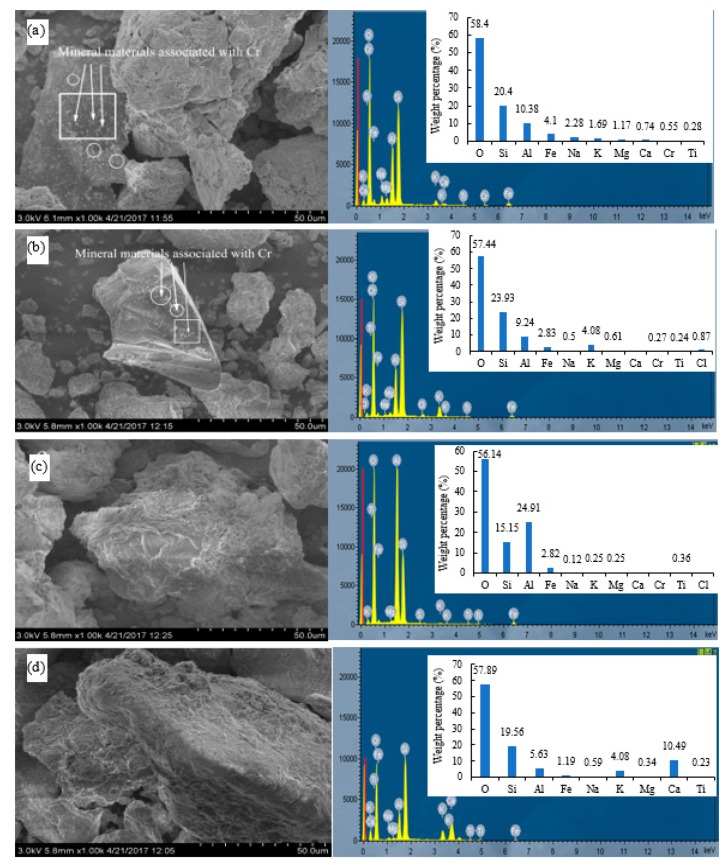
SEM image and EDS of the soils before and after leaching by different acids. (**a**) Original soil, (**b**) soil leached by HCl, (**c**) soil leached by CA, (**d**) soil leached by OX.

The morphology of the test soils was determined using SEM-EDS (Figure 6). The original soil and HCl-treated soil had smooth surfaces with fine grains on them (Figure 6a,b). Cerqueira et al. [37] observed Cu and Cd spotted on the surface of soil particles, and the coexistence of amorphous iron oxides, vermiculite, gibbsite, and organic matter aggregates containing Cu, and vermiculite and gibbsite aggregates containing Cd. Using field emission scanning electron microscopy (FE-SEM) with energy-dispersive X-ray spectroscopy (EDS) techniques, Cu was found on the surface of amorphous iron oxides and associated with schwertmannite [38]. Fe-rich spherules and irregular particles were commonly observed particles in technogenic magnetic particles in industrially contaminated soils, revealing various Fe contents often associated with elevated heavy metal contents [15]. After leaching by organic acids, soils showed significant morphological changes: the surfaces of soil particles became rougher and no fine-grained structures were observed (Figure 6c,d). Considering the chelation of metals by organic acids, it could be inferred that the leaching of Cr and other metal elements in the soil was related to the disappearance of these fine grains. Smaller grains have a higher specific surface area and more reaction sites, so they could be dissolved faster by acids. These results are in accordance with previous findings that heavy metals in soils were combined with certain components and showed a punctate distribution in soil particles [39,40].

Elemental analysis of soils before and after leaching was determined by EDS (Figure 6). The peaks of O, Si, and Al in the original soil samples were higher than those of Ca, Fe, Na, Mg, K, and Cr. This indicates that chemical constituent elements of the original soil microstructure are mainly O, Si, Al, Ca, Fe, Mg, K, Cr, and other trace elements, at least on the surface or near-surface layer. Combined with the results of XRD analysis (Figure 5), it can be inferred that the minerals of the original soil are mainly composed of silicon dioxide or silicate, and the clay minerals were mainly crystalline or non-crystalline silicates composed of Si tetrahedron or Al octahedron and free-state aluminosilicates.

Since organic acids can chelate metals in the process of leaching [7,10,26], the metals in the soil matrix minerals may form chelates with organic acids that could be separated from the soil particle surface. As shown in Figure 6, the distribution and content of elements in the soil after leaching showed a great change. Cr was not detected in the soil surfaces after leaching by CA and OX but was detectable in the original soil and the soil leached by HCl, with a weight percentage of Cr at 0.55% and 0.27%, respectively. Our results suggest that organic acids could effectively desorb Cr in soil particle surface. A remediation strategy using the two organic acids is recommended for Cr-contaminated soil.

The results of EDS analysis are in agreement with the cumulative leaching loss of the metals (Ca, Mg, and Fe) monitored in the leachates (Figure 4). The percentages of O, Si, and Al in all the soils leached by acids were still higher than other elements. In the HCl-leached soil, the weight percentages of Na, Mg, Ca, and Fe decreased, and those of Si and K increased. At the same time, the content of Cl increased after leaching by HCl, which was evidently introduced by the HCl added. In the CA-leached soil, the weight percentages of Fe, Na, K, Mg, and Ca decreased, while the weight percentage of Al increased significantly, which may be due to the formation of insoluble aluminum–citrate complexes. After leaching by OX, the weight percentages of Al, Fe, Na, and Mg significantly decreased, while those of Ca and K were much higher than in original soil, indicating that OX has good retention effects on Ca and K. The elements Ca and K are not only essential for plant nutrition but are also important for maintenance of healthy soil structure [41,42]. Therefore, OX may be more environment-friendly than CA and HCl for soil washing, which is of great importance for subsequent applications of the remediated soil in plant growth and production [43].

### 3.6. XPS Analysis

**Figure 7:**
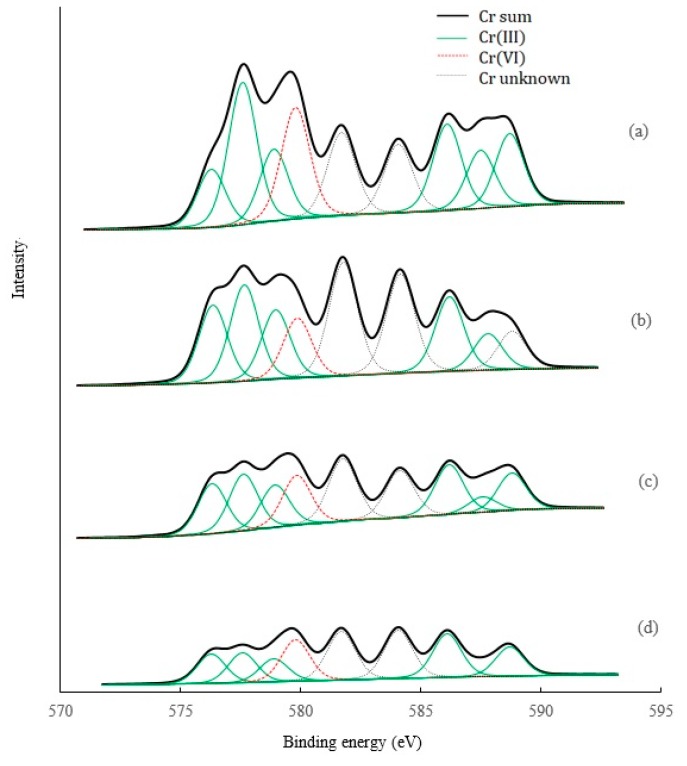
XPS analysis of Cr species in the soils before and after leaching by different acids. (**a**) Original soil, (**b**) soil leached by HCl, (**c**) soil leached by CA, (**d**) soil leached by OX.

XPS analysis provides the information on oxidation states of the Cr elements before and after leaching (Figure 7). The results showed that various chemical states of Cr occurred in the original soil, including Cr(III), Cr(VI), and some other unknown states. Based on the height and area of the peaks, Cr(III) represented the highest proportion. After acid leaching, the heights and areas of all the peaks decreased, suggesting that these acids were effective for removal of all the states of Cr. This is also confirmed by the Cr atomic concentrations based on XPS analysis, with a decreasing order of the original soil (1.25%) > HCl leached soil (1.03%) > CA leached soil (0.83%) > OX leached soil (0.57%). However, compared to HCl, OX, and CA, this showed a significantly higher removal for total, trivalent Cr, and hexavalent Cr. These results suggest that chelation through ligand complexing of Cr(III), in addition to acid dissolution, may be the main mechanisms responsible for Cr(III) removal by organic acids. The main Cr(VI) compound based on the XRD analysis was water-soluble MgCrO_4_, which may explain the effective removal of Cr(VI) by all three acids. HCl has a stronger acidity, but showed no higher removal for Cr(VI) than OX and CA, indicating other different mechanisms such as competitive desorption, or reduction of Cr(VI) to Cr(III) and the subsequent chelation of Cr(III) may occur in Cr(VI) removal by OX and CA.

Previous studies have shown OX to have a significant role in both Cr(VI) reduction and Cr(III) complexation [14,44,45]. CA also can increase the reduction of Cr(VI) and the subsequent formation of soluble Cr(III)-citrate [45], and solubilize the Cr(III) by complexation and desorb the Cr(VI) by competition for surface sites [24]. These mechanisms are probably involved in the removal of Cr, as well as Fe, Mn, Ca, and Mg, in our present study. Evidently, these mechanisms are not involved in Cr-leaching by HCl. Furthermore, compared to CA, OX is a strong acid, and OX of the same quality can provide more ligands (-COOH). These facts can explain why OX possesses a higher removal ability than CA.

Unsurprisingly, acid leaching has several disadvantages, such as disturbing soil structure, causing losses of soil nutrients and increased acidity, and generating a large amount of leachate wastewater. Compared to the remediation techniques based on immobilization technology that cannot remove metals from the contaminated sites, acid leaching is one of the few remediation techniques that is efficient in extracting metals from the soils. For those highly contaminated sites where toxic metals are required to be separated from the soil, acid washing may be an ideal alternative. Particularly, compared to HCl, OX as a biodegradable organic acid leached more Cr but caused less acidity, suggesting a potential in the leaching remediation of Cr-polluted soil.

## 4. Conclusions

Acid leaching could decrease soil pH, causing soil acidification. OX leached much more Cr than CA and HCl, indicating that OX can be used in leaching remediation of Cr-contaminated soil. Ca, Mg, Mn, and Fe were also leached by the three acids, and in most cases, correlated positively with Cr. Results from XRD, SEM-EDS combined with XPS techniques confirmed that Cr mainly interacted with soil minerals and showed a punctate distribution on soil particle surfaces. Soil mineralogical and morphological properties were significantly changed after leaching by acids. In the process of acid leaching, clay minerals, primary silicate minerals, and carbonate minerals in the soil would be decomposed, so that Cr and other metal elements (Ca, Mg, Mn, and Fe) were released into leachate. In addition to the acid dissolution, the ligand complexation of Cr(III), competitive desorption, and reduction of Cr(VI) may represent the main mechanisms underlying Cr leaching by OX and CA. The concomitant removal of other non-targeted metals occurred during the leaching remediation of Cr-contaminated soil, leading to loss of soil mineral nutrients and fertility. Overall, OX leached more Cr but less Ca, and produced smaller soil acidification, and thus can function as an effective and environment-friendly extractant in the leaching remediation of Cr-contaminated soil.

## Figures and Tables

**Table 1:** Physical and chemical properties of the soil.

Soil Property	Value
Soil type	Alfisols (USDA soil taxonomy)
Soil texture	Sandy loam
Soil bulk density (g/cm^3^)	1.36
CEC (cmol/kg)	2.62
pH	10.25
Particle distribution 0.075–2 mm (%)	40.1
OM (%)	5.7
Cr(VI) (mg/kg)	328 ± 21
Total Cr (mg/kg)	3389 ± 169
Total Ca (mg/kg)	17,253 ± 862
Total Mg (mg/kg)	7209 ± 360
Total Fe (mg/kg)	25,263 ± 1268
Total Mn (mg/kg)	546 ± 30

## Supplementary Materials

The following are available online at https://www.mdpi.com/1660-4601/16/15/2771/s1, Figure S1. Correlation between Cr and other metal concentrations in the leachates of HCl (a), CA (b) and OX (c). (ns not significant; * correlation is significant at *p* < 0.05; ** correlation is significant at *p* < 0.01).
