# Supplementary material for: Removal of Chromium from a Contaminated Soil Using Oxalic Acid, Citric Acid, and Hydrochloric Acid: Dynamics, Mechanisms, and Concomitant Removal of Non-Targeted Metals
Source: Int J Environ Res Public Health. 2019 Aug 2;16(15):2771. doi: 10.3390/ijerph16152771 (PMC6696345; doi:10.3390/ijerph16152771)
Supplement: Supplementary file 1 [file ijerph-16-02771-s001.pdf]

Supplementary file

(a)

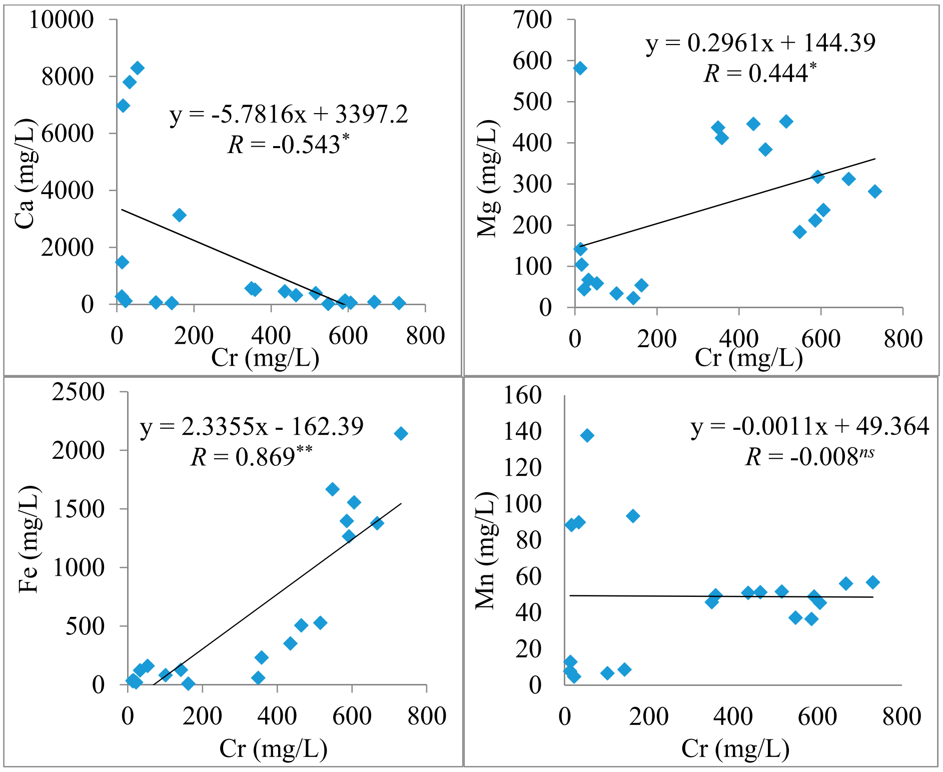

(b)

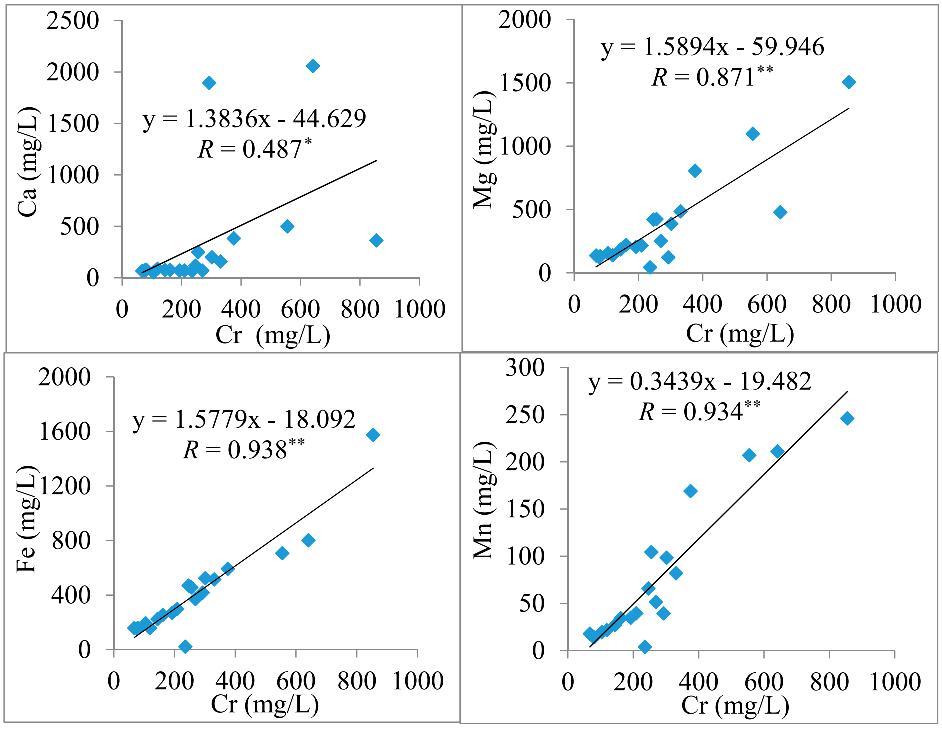

(c)

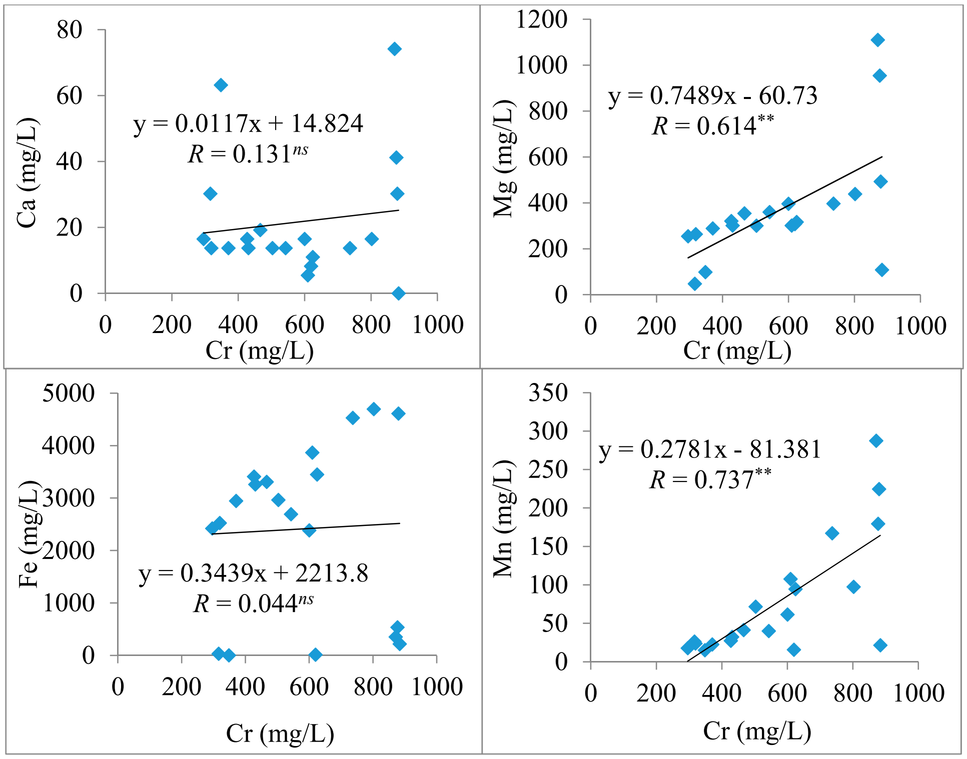

Figure S1. Correlation between Cr and other metal concentrations in the leachates of HCl (a), CA (b) and OX (c). (*ns* not significant; * correlation is significant at $p < 0.05$; ** correlation is significant at $p < 0.01$).
